# Flexible neuronal network simulation framework using code generation for NVidia^®^ CUDA™

**DOI:** 10.1186/1471-2202-12-S1-P239

**Published:** 2011-07-18

**Authors:** Thomas Nowotny

**Affiliations:** 1 Informatics, University of Sussex, Falmer, Brighton, UK

## 

Simulating large scale computer models of brain structures with spiking neuronal networks has become increasingly popular and feasible with the advent of general purpose computing on graphical processing units (GPGPU). Modern graphics cards, such as the NVidia^®^ range supporting the common unified device architecture (CUDA™) provide massively parallel computing architectures for this purpose. Earlier GPU implementations of neural networks, including my own earlier work [1,2], were customized for specific models, and optimized and tested with specific hardware. Recently, more general spiking neuronal network simulators have been developed [3,4] that allow the definition of the network connectivity and neuron- and synapse parameters at runtime. However, the simulators are still quite specific in using a single neuron model (typically Izhikevich neurons), synapse model (typically stateless synapses with delay) and have been tested for a typical model type and on specific GPU hardware.

In this work I present a framework of semi-automated code generation for simulating neuronal networks on GPU hardware. Using code generation to build a specific simulator engine for each individual network model has important advantages: (i) The simulator system can provide a large choice of different neuron and synapse models for use in simulations without creating any overheads or performance losses in the actual simulation code. It also allows the inclusion of user-defined models without the necessity for a user to understand the GPU code. (ii) The generated simulator software can be optimized for the available GPU hardware and for the structure of the specific model. (iii) The framework is intrinsically extensible: New GPU optimization strategies can be added and strategies of existing simulators can be included in the generated code for situations where they are effective. An embryonic beta version of such a framework has been built and optimized for simulating neuronal networks with an anatomical structure (separate neuron populations that are densely connected), building on our earlier work on neuronal network models [1,2] of the olfactory system of insects [5,6].

The prototype framework consists of a C++ source library that generates CUDA kernels and runtime code according to a user-specified neuronal network model. Unlike existing simulators [5,6], it exploits that most parameters of neuronal network simulations are known at compile time and do not change during a simulation. These parameters are hard-coded into the CUDA kernels saving valuable register and shared memory space. In practice, the system is used by defining a neuronal network in a C++ class and compiling and executing the code generation software. The generated C++/CUDA code is compiled with user-side simulation code into a lean stand-alone executable. I have tested the system on an NVidia Quadro FX 5800 device hosted in a PC with Intel Xeon quad core CPU with 8MB cache and 12 GB RAM. I have observed variable GPU versus CPU speedups between none and 76x. The peak observed spike delivery per second was 2.4 billion and the largest simulated network had about 1 million neurons and 635 million synapses (limited by the 4GB device memory on the FX 5800). In the future I will extend the system with additional model elements, optimizations and improved API for use by the CNS community.
